# CDCA5 overexpression is an Indicator of poor prognosis in patients with hepatocellular carcinoma (HCC)

**DOI:** 10.1186/s12885-018-5072-4

**Published:** 2018-11-29

**Authors:** Yunhong Tian, Jianlin Wu, Cristian Chagas, Yichao Du, Huan Lyu, Yunhong He, Shouliang Qi, Yong Peng, Jiani Hu

**Affiliations:** 1 Department of General Surgery, Nanchong Central Hospital, The second Clinical College of North Sichuan Medical College, Nanchong, 637000 Sichuan China; 2 Department of Radiology, Affiliated Zhongshan Hospital of Dalian University, 116011, No.6 of JieFang Road, Dalian, China; 3 Department of Radiology, Wayne State University, Detroit, MI 48201 USA; 4 Department of Hepatobiliary Surgery of the Affiliated Hospital, Southwest Medical University, Luzhou, Sichuan China; 5 The Sino-Dutch Biomedical and Information Engineering School of Northeastern University, Shenyang, China

**Keywords:** CDCA5, Prognosis, Hepatocellular carcinoma

## Abstract

**Background:**

Accurate and early prognosis of disease is essential to clinical decision making, particularly in diseases, such as HCC, that are typically diagnosed at a late stage in the course of disease and therefore carry a poor prognosis. CDCA5 is a cell cycle regulatory protein that has shown prognostic value in several cancers.

**Methods:**

We retrospectively evaluated 178 patients with HCC treated with curative liver resection between September 2009 and September 2012 at Nanchong Central Hospital in Nanchong, Sichuan Province, China. Patients were screened for their CDCA5 expression levels and assigned to either the high or low expression group. Patient demographics, comorbidities, clinicopathologic data, such as tumor microvascular invasion status and size, and long-term outcomes were compared between the two groups. The effect of CDCA5 on the proliferation of liver cancer cells was analyzed using in vitro and in vivo assays.

**Results:**

The present study found that increased CDCA5 expression was associated with increased tumor diameter and microvascular invasion in HCC. It was also found that CDCA5 overexpression may be associated with liver cancer cells. Additionally, this study confirmed that CDCA5 expression was increased in HCC tissue versus normal liver tissue, that CDCA5 expression was associated with decreased survival and that CDCA5 knockdown using shRNA led to cell cycle arrest in the G2/M phase.

**Conclusions:**

These findings suggest that CDCA5 expression is associated with poor prognosis in patients with hepatocellular carcinoma.

## Background

Although the incidence of hepatocellular carcinoma (HCC) in China has decreased, it remains a serious health issue and a major cause of morbidity and mortality. In 2015, the estimated total incidence of HCC in China was 466,100 patients, of which 343,700 were male and 122,300 were female. The estimated mortality was 422,100 people, with 310,600 males and 111,500 females affected [1, 2]. Accurate prognosis is essential to optimizing disease outcomes, particularly in regard to selecting the most effective treatment. This is especially true for diseases, such as hepatocellular carcinoma, for which curative options are largely surgical and therefore carry their own mortality risks [3]. As such, prognostic and predictive factors, such as molecular markers, are invaluable and can heavily impact clinical decision making [4].

Cell-division cycle-associated 5 (CDCA5), also known as sororin, is thought to play a critical role in ensuring the accurate separation of sister chromatids during the S and G2/M phases of the cell cycle through interactions with cohesin and cdk1 [5, 6]. CDCA5 has also been shown to interact with ERK as well as cyclin E1, a critical regulator of the G1/Smitotic checkpoint [5–7]. Recent studies have correlated the expression of CDCA5 with tumorigenesis and tissue invasion in several cancers, including oral squamous cell cancer, non-small cell lung cancer, urothelial cell carcinoma, and gastric cancer [7–9].

The objective of this study was to confirm the prognostic value of CDCA5 expression levels in HCC and to shed light on tumor characteristics associated with CDCA5 expression. This was accomplished by(1)examining the effects of CDCA5 expression on microvascular invasion and tumor diameter in HCC using resected specimens from our patient population, (2) determining differences in CDCA5 expression levels between HCC, normal hepatic, and peri-tumoral hepatic tissues,(3)confirming the effects of CDCA5expression on the postoperative survival rate of HCC patients by performing a retrospective analysis of our patient population, and (4) confirming the effects of CDCA5 expression on viability, proliferation, and apoptosis in hepatoma cell lines using knockdown and gene amplification in vitro and in a mouse model.

## Methods

### Clinical data

A retrospective analysis of the clinical data from 178 patients who underwent curative liver resection for HCC between September 2009 and September 2012 was performed. All procedures were performed by the department of hepatobiliary surgery at Nanchong Central Hospital in Nanchong, Sichuan Province, China. Patients’ age, gender, preoperative Child-Pugh score, serum AF*P* value, platelet count, HBV-DNA status, MELD score, and antiviral treatment use were collected. Postoperative pathological details, including liver cirrhosis, tumor diameter, number of tumors, minimum distance from the tumor margin, anatomical liver resection, intraoperative blood transfusion, tumor capsule information, microvascular invasion, Edmondson-Steiner grade, and postoperative complications, were also recorded and obtained. This study was approved by the Ethics Committee of the Second Clinical Medical College of North Sichuan Medical College.

### Materials

An anti-CDCA5 monoclonal antibody was purchased from Abcam. The lentiviruses pSICOR and pCDH-CMV-MCS-EF1-Puro, HepG2 cell lines, DNA gel recovery kits, and LATaq were purchased from Hualianke. Endotoxin plasmid extraction kits were purchased from OMEG. T4 DNA ligase and the endonucleases Xho I, BamH I, and Xba I were purchased from NEB. DMEM was purchased from HyClone. Fetal bovine serum and penicillin were purchased from Gibco. PBS, 0.25% trypsin, and propidium iodide were purchased from Bioswamp. Lipofectamine 2000 was purchased from Invitrogen. A Lentiviral Packaging Kit was purchased from Shanghai Shengsheng Biotechnology Co., Ltd. RibonucleaseA (RNaseA) was purchased from TaKaRa. The DH5α strain was obtained from existing laboratory stocks.

### Cell culture

SMMC-7721, HepG2, and Huh-7 cells were cultured in DMEM containing 10% fetal bovine serum and 1% penicillin at 37 °C in a 5% CO2 incubator. The cells were then digested in 0.25% trypsin and subcultured.

### Experimental animals

Twelve SPF grade BALB/C-nu/nu nude mice, 4 to 6 weeks old and weighing 18–20 g, were purchased from Beijing Huafu Kang Biotechnology Co., Ltd. They were raised in an SPF animal room.

### Immunohistochemical staining

The specific steps of anti-CDCA5 monoclonal antibody immunohistochemical staining were performed according to the kit instructions. The main staining procedures were as follows: paraffin section dewaxing, hydration, 3% H2O2 incubation to eliminate endogenous peroxidase activity, high pressure antigen retrieval, normal goat serum blocking, primary antibody (CDCA5 monoclonal antibody) incubation overnight at 4 °C, and 3 PBS washes. The secondary antibody (goat anti-rabbit IgG antibody-HRP) was then added. After the incubation, the tissues were rinsed 3 times with PBS. DAB staining, hematoxylin counter staining, alcohol dehydration, transparent xylene incubation, and neutral gum sealing were performed. Prepared samples were observed via microscopy.

### Plasmid construction

Total RNA was extracted from human hepatoma tissue and reverse transcribed into cDNA. This cDNA was used as a template to amplify the CDCA5 gene by using primers specific for CDCA5 (CDCA5-F: GCTCTAGATGTCTGGGAGGCGAACGC, CDCA5-R: CGGGATCCTCATTCAACCAGGAGATCA). This CDCA5 gene and the plasmid pCDH-CMV-MCS-EF1-Puro were double-digested with Xba I and BamH I, and the CDCA5 gene was subsequently ligated in to the plasmid pCDH-CMV-MCS-EF1-Puro using T4 DNA ligase. This construct was then transformed into competent DH5α cells. Positive clones were used to expand the cultures, and the plasmids were extracted using an endoviral plasmid extraction kit. The *CDCA5* overexpression plasmid with the correct sequence was named pCDH-CDCA5. Based on the GenBank sequence of CDCA5 (NM_080668.3), 3 shRNA interference sequences were designed using the shRNA design program. In addition, Xho I and BamH I restriction sites were introduced into the 5′ and 3’ends of the shRNAs, and the double-stranded DNA oligo containing the interference sequence was synthesized according to this design. The pSICOR vector and the synthesized interference sequence were double-enzyme linearized by T4 DNA. The construct was then ligated into the PSICOR vectorbyT4DNA ligaseand transformed into competent DH5_α_cells. The positive clones were picked and expanded. The plasmid was extracted using an endotoxin plasmid extraction kit, and the correctly sequenced CDCA5 interference plasmid was named pSICOR-shCDCA5. Plasmids were sent to Shanghai Huajin Biotechnology Co., Ltd. for sequencing.

### MTT assay

HepG2 cells were seeded into petridishes and infected with each plasmid. The HepG2 normal cells group, CDCA5 low expression group, and CDCA5 overexpression group logarithmic phase cells were collected, and the cell suspension was distributed into a 96-well plate with 180 μL per well (1~ 5 × 10^3^ cells/well) in triplicate, and 100 μL culture solution was used as the blank control. The cells were incubated at 37 °C for 12 h, 24 h, 48 h, and 72 h. Then, 20 μl MTT solution (5 mg/ml) was added to each well, and then the cells were cultured for 4 h. Then, 150 μl DMSO solution was added to each well after the supernatant was discarded. The plate was shaken at low speed for 10 min to fully dissolve the crystals. The 490 nm absorbance of each well was measured in an enzyme-linked immunosorbent assay.

### Colony formation

Cells were trypsinized, washed, diluted 1/10 and seeded into10 cm dishes after transfection. Cells were grown for 2 weeks, after which colonies were stained with Coomassie blue (0.1% Coomassie blue, 30% methanol, 10% acetic acid) and counted.

### Flow cytometry detection of the cell cycle

The CDCA5 low expression group cells, CDCA5 overexpression group cells and negative control HepG2 cells were digested with 0.25% trypsin, transferred to flow cytometry tubes, and centrifuged at 1000 rpm for 5 min. Each precipitate was suspended in 300 μl PBS solution containing 10% fetal bovine serum and then transferred into a clean 1.5 ml centrifuge tube. Then, 700 μl anhydrous ethanol was added, and the cells were fixed in a refrigerator at − 20 °C for at least 24 h. Flow cytometry was performed to quantify the number of cells in each stage of the cell cycle.

### Tumorigenicity experiment in nude mice

Six mice were assigned to each group. HepG2 cells with low expression of CDCA5 and the negative control cells were digested with 0.25% trypsin, and the cell numbers were counted. The cell concentration was adjusted to 1 × 10^7^ cells/ml with serum-free medium. A 0.2 ml cell solution was subcutaneously injected into each mouse using both HepG2 cells with low expression of CDCA5 and negative control cells. We observed the survival of the nude mice and the growth of the tumors. All nude mice were sacrificed by cervical dislocation on the 30th day, and the tumors were removed. The average weights of the tumors in each group were calculated.

### Statistical analysis

Continuous data are shown as the mean ± standard deviation. Comparisons of continuous data were carried out using Student’s t test or the Mann-Whitney U test. Multiple groups of measurement data were compared using one-way ANOVA. Categorical variables were compared using the chi-square test or Fisher’s exact test as appropriate. The Kaplan-Meier method was used to estimate survival probabilities, which were compared using the log-rank test. All statistical tests were two-tailed, and a *P* value < 0.05 was considered significant. Statistical calculations performed using SPSS software (IBM version 22.0, NY) [10].

## Results

### Clinical and pathological features of patients

#### Clinical characteristics

After a review of the inclusion and exclusion criteria, 178 patients were included in the study. The average ages of the patients in the CDCA5 high expression group and the CDCA5 low expression group were 50.70 ± 10.02 and 49.54 ± 9.64 years, respectively. The average follow-up time was 40.2 months. There were no significant differences in clinical features between the two groups in terms of gender, liver function, Child-Pugh score, alpha fetoprotein (AFP) level, platelet (PLT) count, antiviral therapy use, hepatitis B virus DNA (HBV-DNA) status, or Model-End-Stage liver disease (MELD) score (Table 1).

**Table 1:** The clinical and pathologic characteristics of the included patients

Variable	Patients (178)	CDCA5 overexpression group	Low CDCA5 expression group	Odds Ratio	*P* value
Liver cirrhosis, n(%)	128 (71.91)	63 (70.79)	65 (73.03)	0.895	0.739
Tumor diameter, median (range)	5.88 ± 3.83	6.67 ± 3.97	5.11 ± 2.42	0.890	0.006
Tumor number, (%)				0.624	0.189
1	156 (87.64)	82(92.13)	74 (83.15)		
2	16 (8.99)	5 (5.62)	11 (12.36)		
≥ 3	6 (3.37)	2(2.25)	4 (4.49)		
Tumor margin,< 2/≥2 cm	45/178	22/67	23/66	0.942	0.897
Anatomic/nonanatomic liver resection	73/105	38/51	35/54	1.105	0.648
Intraoperative blood transfusion, Yes/No	27/151	15 / 74	12 / 77	1.301	0.531
Tumor capsule, complete/incomplete	88/90	39 / 50	43 / 46	0.834	0.548
Microvascular invasion, present/absent	44 / 134	28 / 61	16 / 73	2.094	0.037
Edmondson-Steiner Grade I / II / III / IV	33 / 78 / 65 /2	12 / 37 / 38 / 2	21 / 41 / 27 / 0	0.608	0.089

#### Pathological features

Patients with CDCA5 overexpression had larger tumor diameters and a higher incidence of microvascular invasion compared with patients with decreased CDCA5 expression. The odds of microvascular invasion in the high CDCA5 expression group were over twice that in the low expression group. There were no significant differences in the proportion of cirrhotic patients, number of tumors, tumor capsule characteristics, or the degree of differentiation between the two groups (Table 1).

### The expression of CDCA5 in HCC

**Fig. 1:**
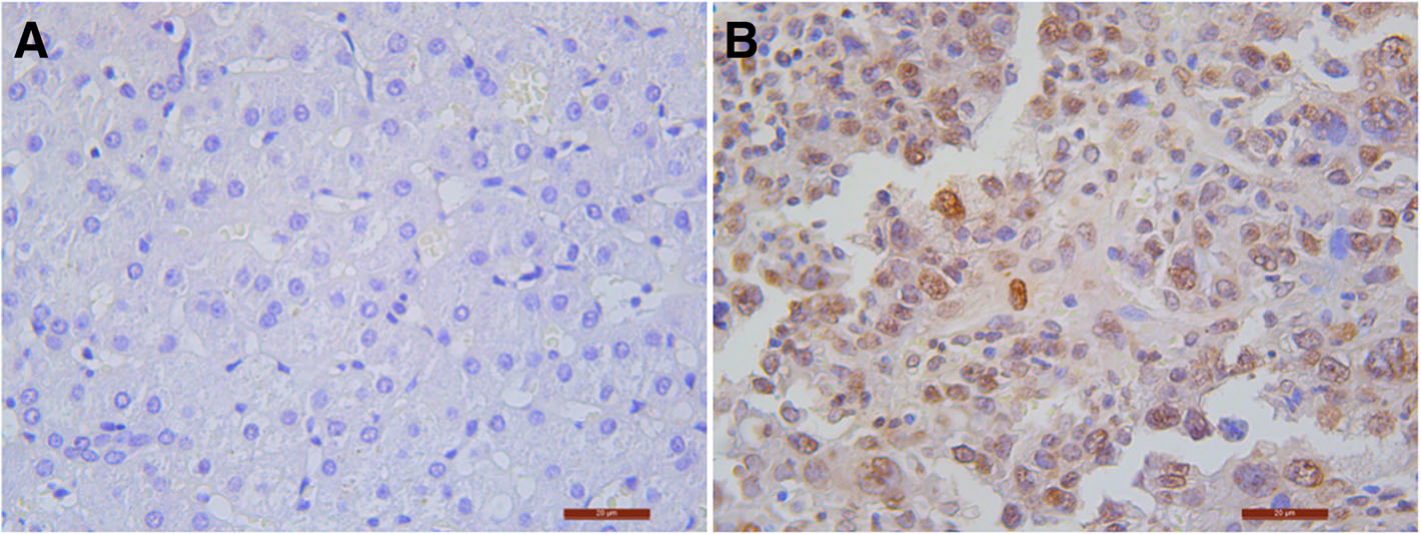
Immunohistochemistry shows *CDCA5* expression in normal liver tissue (**a**) and liver cancer tissue (**b**). (DAB staining, hematoxylin counterstain, magnification: 400 x)

**Fig. 2:**
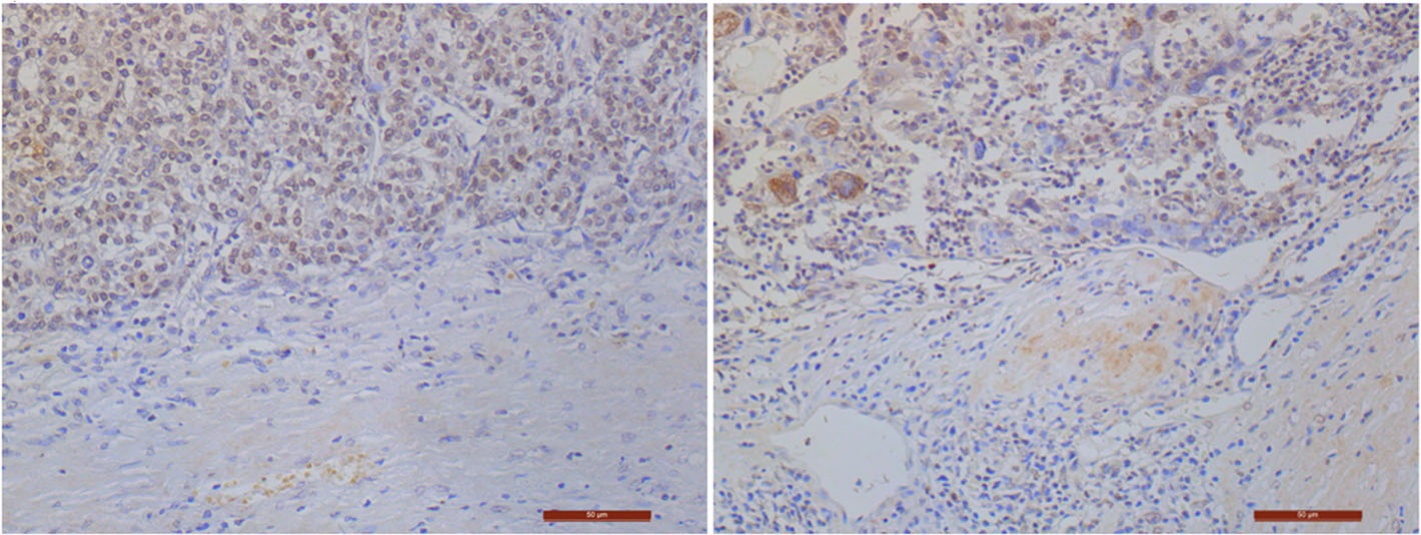
Immunohistochemistry shows CDCA5 expression in tumor tissue and peritumor tissue samples. (DAB staining, hematoxylin counterstain, magnification: 200 x)

We used the median of the semiquantitative CDCA5 expression data from the 178 HCC tissue samples as a cutoff point for high and low expression of CDCA5. There were 89 patients with high expression of CDCA5 and 89 patients with low expression of CDCA5. Compared to normal liver tissue, HCC tissue exhibited significantly increased expression of CDCA5 (*P* < 0.05). (Figs. 1, 2).

### Survival analysis

#### Effect of the CDCA5 expression level on the postoperative survival rate of HCC patients

**Fig. 3:**
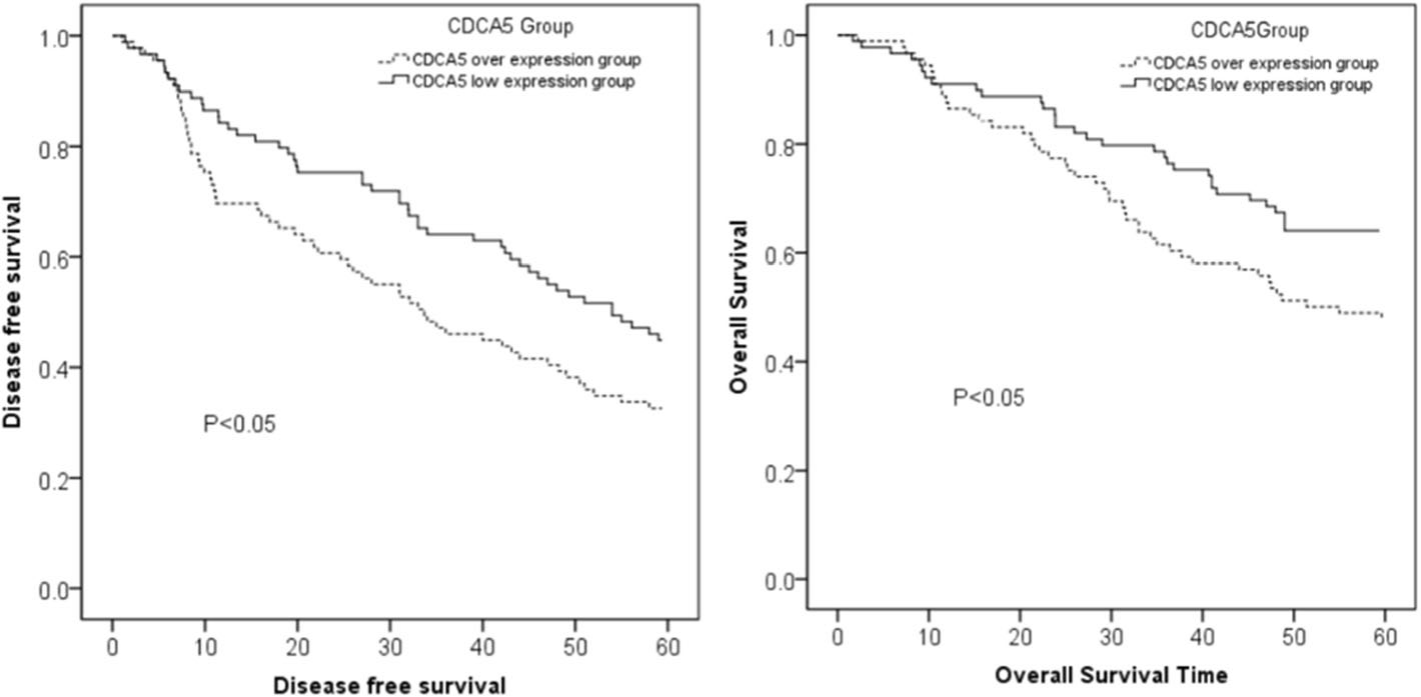
The survival curves for the CDCA5 overexpression group and the low CDCA5 expression group

The 1-, 3-, and 5-year disease-free survival rates in the CDCA5 overexpression group were 69.7, 46.1, and 32.6%, respectively. The 1-, 3-, and 5-year overall survival rates in the CDCA5 overexpression group were 86.5, 61.5, and 47.8%, respectively. The 1-, 3-, and 5-year disease-free survival rates in the CDCA5 low expression group were 84.3, 64.0, and 44.9%, respectively. The 1-, 3-, and 5-year overall survival rates in the CDCA5 low expression group were 89.9, 76.4, and 64.0%, respectively. The survival rates of the patients in the CDCA5 overexpression group were worse than those of the patients in the CDCA5 low expression group (*P* < 0.05) (Fig. 3).

### Expression of CDCA5 in HCC cell lines

**Fig. 4:**
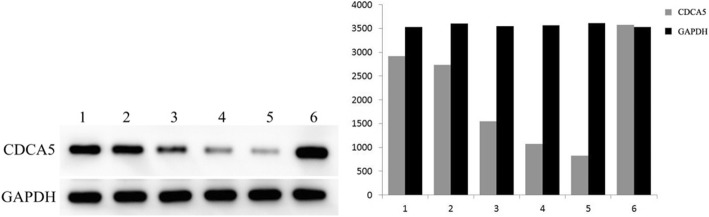
CDCA5 protein expression after shRNA interference or overexpression by plasmid liposomes encoding CDCA5. 1. Control group; 2. Negative control group; 3.shCDCA5–1 interference group; 4. shCDCA5–2 interference group; 5. shCDCA5–3 interference group; 6. CDCA5 overexpression group

We selected the SMMC-7721, HepG2, and Huh-7 HCC cell lines and detected the expression of CDCA5 protein by Western blotting. HepG2 cells had the highest expression of CDCA5 and were selected for the knockdown experiment. SMMC-7721 cells had the lowest expression of CDCA5 and were selected for the overexpression experiments. Plasmids that knocked down and overexpressed CDCA5 were constructed, and Western blotting was used to detect changes in CDCA5 expression after knockdown and overexpression (Fig. 4). Finally, we chose to use the pSicoR-shCDCA5–3 and pCDH-CDCA5 plasmids in the following experiments.

### MTT assay

The proliferation rates were detected by an MTT assay after CDCA5 knockdown in HepG2 cells and CDCA5 overexpression in SMMC-7721 cells. The growth inhibition rates of the HepG2 cells in the knockdown group after transfection with pSicoR-CDCA5–3 were 8.40 ± 2.07%, 14.10 ± 0.53%, 65.97 ± 0.58% and 70.10 ± 1.04% at 12, 24, 48 and 72 h, respectively(*P* < 0.05). When the transfection time was prolonged, the growth rate of the HepG2 cells in the *CDCA5* knockdown group was significantly decreased. The survival rates of the SMMC-7721 cells in the overexpression group were 102.83 ± 1.56%, 116.23 ± 1.01%, 128.93 ± 0.95% and 130.03 ± 0.35% at 12, 24, 48 and 72 h, respectively(*P* < 0.05). When the transfection time was prolonged, the survival rate of the SMMC-7721 cells in the CDCA5 overexpression group was significantly increased.

### Colony formation assay

The colony formation rate was 16.49 ± 1.75% in the CDCA5 knockdown group compared to 32.17 ± 3.25% in the negative control group. The quantity of clones formed in the CDCA5 low expression group was less than that of the negative control group (*P* < 0.05)(Fig. 5.1). The proliferation ability of HepG2 cells was attenuated after CDCA5 knockdown. After CDCA5 overexpression, the colony formation rate was 51.93 ± 3.46%, while it was 34.57 ± 4.86% in the negative control group. The quantity of clones formed in the CDCA5 overexpression group was greater than that of the negative control group (P < 0.05). The proliferation ability of SMMC-7721 cells was increased after CDCA5 overexpression (Fig. 5.2).

**Fig. 5:**
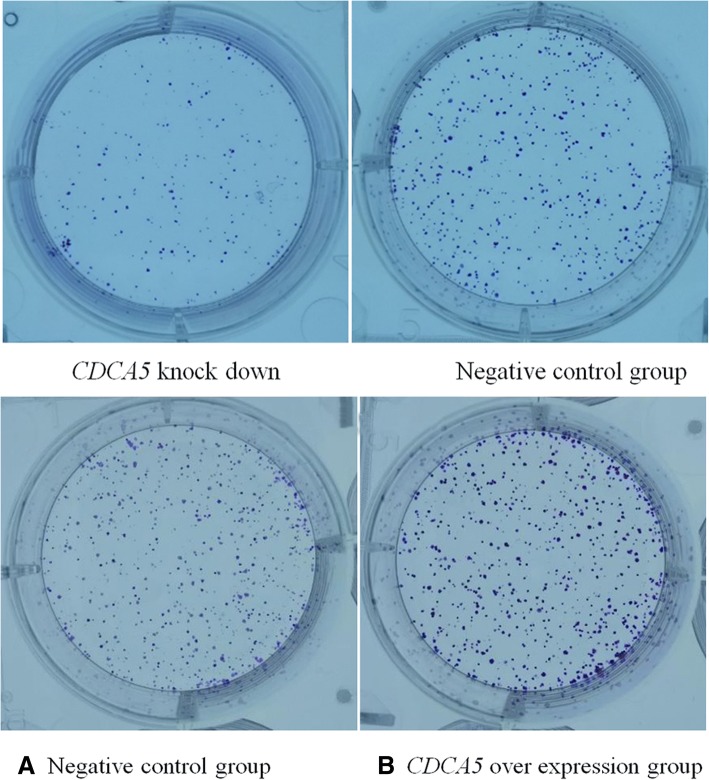
1 HepG2 cell colony formation assay. 2 SMMC-7721 cell colony formation assay

### Flow cytometry

Flow cytometry was used to analyze the cell cycle phases of the HepG2 cells with low CDCA5 expression, SMMC-7721 cells with CDCA5 overexpression, and negative controls. The accumulation of G2 phase cells was significantly increased in the CDCA5 low expression group compared with the negative controls. There were no significant differences between the CDCA5 overexpression group and the negative control group with regard to cell distribution in the cell cycle phases. The results suggested that the inhibition of HepG2 cell growth was associated with arrest in the G2 phase after CDCA5 knockdown (Fig. 6.1, 6.2, Table 2).

**Fig. 6:**
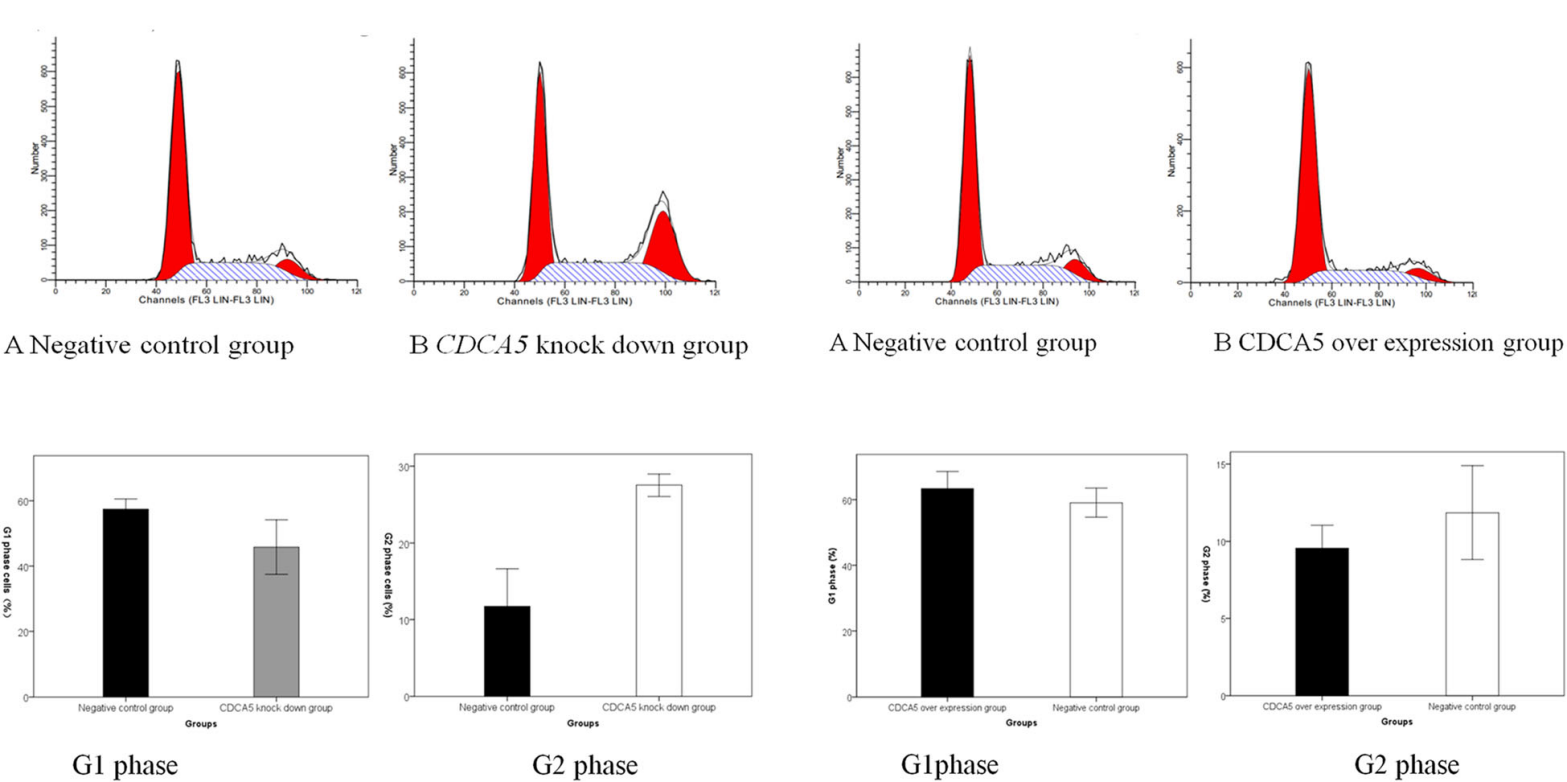
1 Flow cytometry cell cycle analysis of HepG2 cells after CDCA5 knockdown. 2 The histogram shows the proportions of cells in the G1 and G2 phases after the knockdown of CDCA5 expression by pSicoR-CDCA5–3 for 48 h. 3 Flow cytometry was used to detect the changesin the cell cycle parameters of SMMC-7721 cells with CDCA5 overexpression. 4 The histogram shows the proportions of cells in the G1 and G2 phases after the overexpression of CDCA5 by pCDH-CDCA5 for 48 h compared to those after the transfection of the negative controls for 48 h

**Table 2:** The cell cycle distribution of HepG2 cells in the low expression group and control group

Group	G1 (%)	S (%)	G2/M (%)
Control group	57.44 ± 1.25	29.92 ± 0.22	11.73 ± 1.97
Low CDCA5 expression group	45.81 ± 3.37	27.37 ± 2.78	27.53 ± 0.58
P value	0.005	0.189	< 0.001

Changes in cell cycle profiles of SMMC-7721 cells after the overexpression of CDCA5.

There were slightly fewer cells in the G2/M phase among the SMMC-7721 cells that overexpressed CDCA5 from pCDH-CDCA5 than among the negative control SMMC-7721 cells. However, these results were statistically insignificant (Fig. 6.3, 6.4, Table 3).

**Table 3:** The cell cycle distribution of SMMC-7721 cells in the over expression group and control group

Groups	G1 (%)	S (%)	G2/M (%)
Negative control group	59.04 ± 1.76	29.38 ± 1.21	11.86 ± 1.22
CDCA5 over expression group	63.25 ± 2.10	27.97 ± 4.05	9.55 ± 0.60
P value	0.057	0.595	0.050

### Tumor formation experiments in nude mice

In the control group injected with HepG2 cells, tumors were detectable on the fifth day. In contrast, in the CDCA5 low expression group, tumors were detectable on the sixth day. On the 30th day after injection, the tumor weights in the control group and the CDCA5 low expression group were 0.89 ± 0.07 and 0.66 ± 0.11 g, respectively. Tumors from the HepG2 group were heavier than those from the low CDCA5 expression group, and the difference between the two groups was statistically significant (*P* < 0.05) (Fig. 7.1, 7.2).

**Fig. 7:**
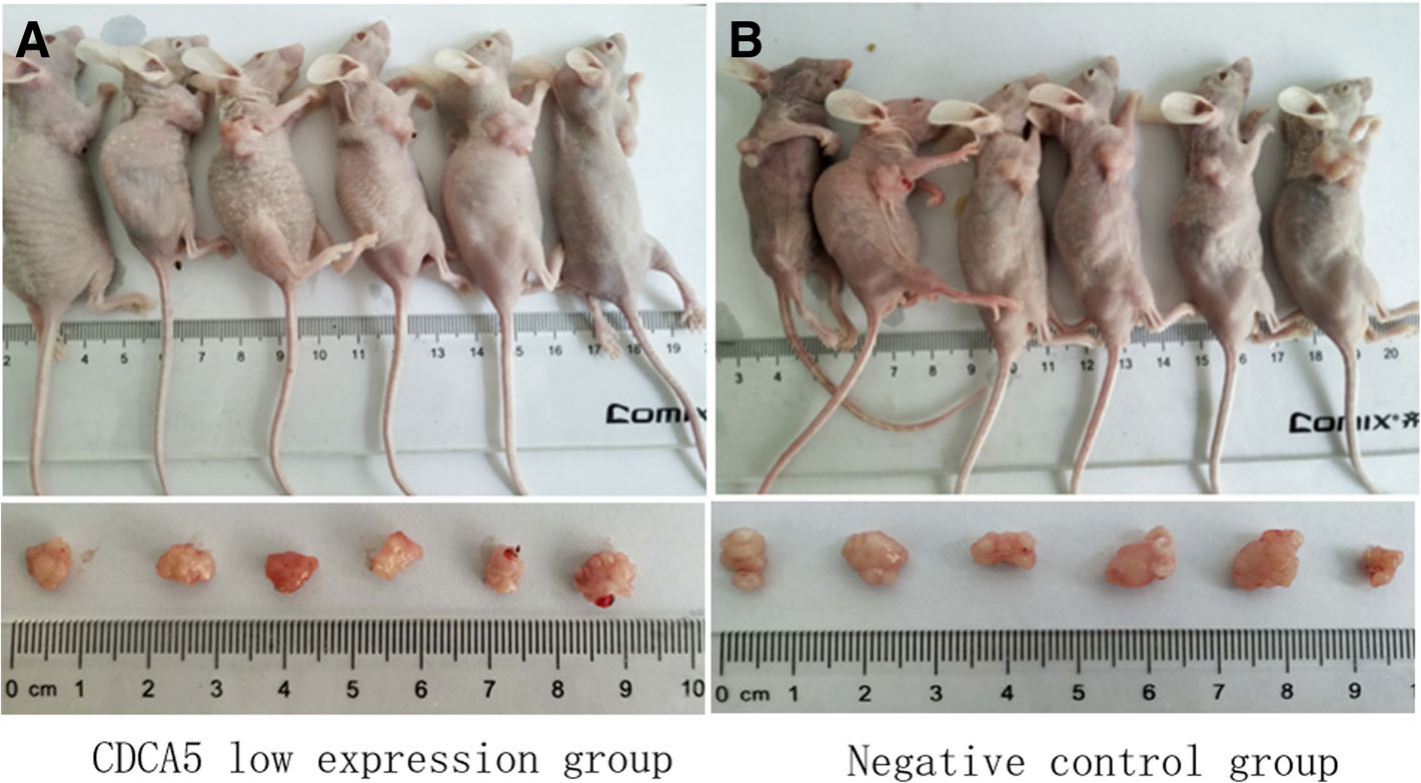
1 Tumorigenicity of HepG2 cells in nude mice. 2 Tumors with different expression levels of CDCA5 formed in nude mice

### Immunohistochemical detection of CDCA5 expression in tumor tissue

**Fig. 8:**
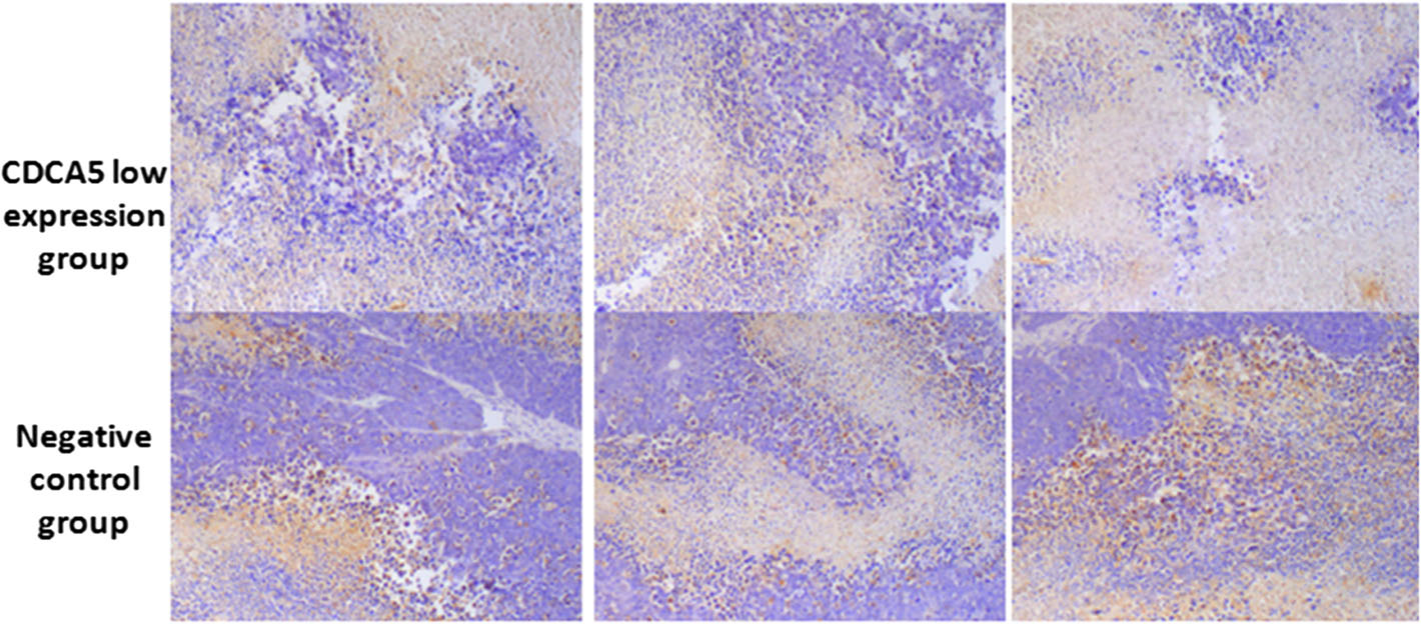
CDCA5 expression in tumors produced by different groups of cells in mice (DAB staining, hematoxylin counterstain, magnification: 200 x)

CDCA5 expression was significantly decreased in the tumor tissue from the CDCA5 low expression group compared with that from the HepG2 group (P < 0.05). (Fig. 8).

## Discussion

HCC is the third leading cause of cancer-related death globally, accounting for over 700,000 deaths per year [2]. The disease carries a high mortality rate because treatments are invasive, frequently requiring liver resection, and patients tend to present with later stage disease as the early stages are asymptomatic. Accurate prognosis is an important step in the management of patients diagnosed with HCC. The main results of this study are as follows: (1) CDCA5 expression is directly correlated with microvascular invasion, (2) CDCA5 expression is directly correlated with tumor diameter, and (3) CDCA5 overexpression is associated with changes in cell cycle parameters as quantified by flow cytometry. In addition, CDCA5 expression was confirmed to be inversely related to disease-free and overall survival in HCC patients. CDCA5 expression was also confirmed to be higher in HCC samples than in normal liver samples. In hepatoma cell lines, CDCA5 expression was confirmed to increase cell viability and proliferation, while CDCA5 inhibition caused decreased rates of proliferation and apoptosis based on colony formation assays, flow cytometry, and nude mouse experiments [11]. These results suggest that CDCA5 is a useful biomarker for prognosis.

The incidence of microvascular invasion was higher in patients with overexpression of CDCA5 (45.90%) than in those with low expression of CDCA5 (21.92%). Although an association between CDCA5 and microvascular invasion in urothelial carcinoma has been shown [11], this is the first study reporting this relationship in HCC. Previous studies have found that microvascular invasion is predictive of survival in patients with HCC, which may explain findings regarding CDCA5 expression and survival [12–14].

This was also the first study demonstrating that CDCA5 expression was directly correlated with tumor diameter. The median tumor diameter in the high CDCA5 expression group was 6.67 ± 3.97 versus 5.11 ± 2.42 in the low CDCA5 expression group (*P* = 0.006). Tumor diameter has been shown to have a significant impact on postoperative survival in HCC patients [13] and is included in multiple liver cancer staging systems, such as the TNM staging system and Milan liver transplantation criteria [15, 16]. Tumor size alone, however, is not sufficient to determine a cancer prognosis. Studies have shown that the biological characteristics of tumors and remnant liver function in patients who have undergone liver resection were more impactful in terms of predicting overall survival [17]. Combining other predictive factors, such as molecular biomarkers and tumor location, with a tumor diameter index is likely to be a better approach to determine prognosis.

This is the first study comparing the effects of different levels of increased CDCA5 expression in CDCA5-positive HCC cells. These results suggest that higher levels of CDCA5 expression in HCC may be associated with liver cancer progression. This is consistent with our hypothesis that CDCA5 expression is associated with more aggressive HCC phenotypes, although these findings were not statistically significant. One possible reason for this is that the SMMC-7721 cell line used for the amplification experiment had lower natural CDCA5 expression than the HepG2 negative controls, such that there was only a modest difference in CDCA5 expression between the two groups. Further experimentation may be helpful in confirming these preliminary findings.

An inverse correlation between CDCA5 expression in HCC and survival was also observed. Specifically, there was a statistically significant 12.3% difference in disease-free survival at 5 years as well as a 16.2% difference in overall survival at 5 years between patients with high CDCA5 expression and patients with low CDCA5 expression (*n* = 178). These results are largely consistent with Shen et al.’s findings, which showed 5-year survival differences between the high and low expression groups of approximately 12% for disease-free survival and approximately 14% for overall survival [11]. Furthermore, CDCA5 expression was higher in tumor tissue than normal hepatic or peritumoral tissue by immunohistochemistry in all 178 samples. The close consistency of our results with those of previous studies supports the idea of a role for CDCA5 in HCC prognosis [7–9, 11].

This study also confirmed that the inhibition of CDCA5 expression in hepatoma cells decreased the rate of proliferation and increased the rate of apoptosis as determined by colony formation assays and flow cytometry, which was consistent with previous studies [11]. The results also support the putative role of CDCA5 in chromatid separation during the G2/M transition [5, 6]. The effect of CDCA5 knockdown on tumor proliferation has been demonstrated in other cancers; in studies of oral squamous cell carcinoma and lung carcinogenesis, it was found that CDCA5 functions as a critical gene in proliferation and progression and that in certain cases, targeting CDCA5may be a useful therapeutic intervention [7, 8]. These findings were further confirmed using nude mouse tumorigenesis experiments. The negative control mice developed larger tumors at 30 days (0.89 ± 0.07 g) than the CDCA5 knockdown mice (0.66 ± 0.11 g). Tumorigenesis was also faster in negative control mice than in the CDCA5 knockdown mice, with tumors being detected on the fifth day in the controls and on the sixth day in the knockdown group. Based on these studies and our results, CDCA5 is not only prognostic but may also be a potential treatment target in HCC.

The limitations of this study include the following. First, this was a pioneering single-center study on the impact of a novel biomarker on disease prognosis, with a total patient population of *n* = 178. Larger, multicenter studies may offer a greater degree of confidence supporting the role of CDCA5 in HCC. Second, the impact of CDCA5 expression in HCC as a screening or prognostic tool on the management of patients with HCC will require further study to determine if use of CDCA5 expression levels should become common practice. Further studies determining the utility of CDCA5 as a prognostic or predictive factor are needed. Last, although well beyond the scope of the present study, the all the molecular pathways in which CDCA5 plays a role have not been defined. Pathways in which CDCA5 has been implicated include the Wnt/β-catenin, RAS/RAF/MAPK, and cyclin/cdk1pathways [7, 9, 11, 18].

## Conclusions

The present study found that CDCA5 overexpression in HCC was correlated with decreased survival, increased microvascular invasion, and increased tumor size in a real patient population. The effects of CDCA5 suppression observed in vitro confirm that it plays an essential role in the dysregulation of cell division. In brief,CDCA5 is a promising novel prognostic factor for patients with hepatocellular carcinoma.

## Abbreviations

AFP: Alpha fetoprotein
CDCA5: Cell-division cycle associated 5
HBV-DNA: Hepatitis B virus DNA
HCC: Hepatocellular Carcinoma
MELD: Model-End-Stage liver disease
PLT: Platelet
